# Urgent health concerns: Clinical issues associated with accidental ingestion of new metal-blade-containing sticks for heated tobacco products

**DOI:** 10.18332/tpc/190634

**Published:** 2024-07-12

**Authors:** Paola Angela Moro, Francesca Maida, Renata Solimini, Lorenzo Spizzichino, Charlotte G.G.M. Pauwels, Elke Pieper, Anne Havermans

**Affiliations:** 1 Poison Control Center of Niguarda Hospital, Milan, Italy; 2 Istituto Superiore di Sanità, National Centre on Addiction and Doping, Rome, Italy; 3 Ministry of Health, Rome, Italy; 4 Centre for Health Protection, RIVM, National Institute for Public Health and the Environment, Bilthoven, the Netherlands; 5 Federal Institute for Risk Assessment (BfR), Unit Product Research and Nanotechnology, Department Chemicals and Product Safety, Berlin, Germany

**Keywords:** heated tobacco product (HTP), ingestion, children, metal blade, abdominal X-rays, endoscopy

## Abstract

**INTRODUCTION:**

Recently, a concerning pattern has emerged in clinical settings, drawing attention to the potential health risks associated with the accidental ingestion, mostly by children, of a new Heated Tobacco Product (HTP) stick, which contains a sharp metal blade inside.

**METHODS:**

Following a webinar of the Joint Action on Tobacco Control 2 project, where data on adverse health incidents related to novel tobacco and nicotine products from EU Member States were presented, the Milan Poison Control Center (PCC) conducted a case series study on the accidental ingestion of blade-containing HTP sticks in Italy, between July 2023 and February 2024. The data in the medical records were analyzed to identify the age distribution, clinical presentation symptoms, performed diagnostic procedures, and medical management.

**RESULTS:**

Overall, 40 cases of accidental ingestion of HTP sticks were identified and are described. A total of 33 (82.5%) children (infants and toddlers, mean age 12.3 ± 3.3 months) were hospitalized. Of these, 29 underwent abdominal X-rays, two children underwent esophagogastroduodenoscopy, and one child suffered from cut injuries to the tonsillar pillar and genian mucosa, requiring anesthesia for fibroscopy. The observed clinical cases associated with new HTP sticks containing a metal blade occurred over just eight months. This issue required the immediate implementation of corrective measures to mitigate health risks. The Ministry of Health issued an alert regarding the dangers related to the accidental ingestion of the stick and imposed more visible warnings on the package.

**CONCLUSIONS:**

It is of the utmost importance to raise awareness among both the general public and medical practitioners to prevent further cases of accidental ingestion of HTP sticks by infants and toddlers, and ensure a prompt and informed response in emergency situations.

## INTRODUCTION

Recently, a concerning pattern has emerged in clinical settings, drawing attention to the potential health risks associated with the accidental ingestion of blade-containing HTP sticks, mostly by infants and toddlers. This new type of stick, designed for use with the new HTP Iqos Iluma, contains a sharp metal blade inside. The metal blade acts as a ‘susceptor’, facilitating the electromagnetic induction process by the device, which in turn heats the tobacco in the stick to temperatures typically below 350^o^C degrees^1^.

A few cases of accidental ingestion of HTP sticks by children have previously been described in the scientific literature^2-4^. Since 2016, the global tobacco retail value has been driven by the growth of emerging tobacco products, including HTPs, particularly in high-income countries^5^.

Indeed, HTP use has increased worldwide^6^. In Europe, HTPs are especially used by younger people and current and former smokers^7,8^. HTPs were first introduced to the Italian market in November 2014^9^. In just seven years, they have become the second most popular tobacco product (after conventional cigarettes and preceding roll-your-own tobacco), with an estimated market share of 18% in 2023. According to the national report on tobacco use in 2023^10^, the exclusive HTP users in Italy were 3.7% of the population, while the dual users (HTPs and conventional cigarettes) were approximately 86%. With respect to the younger population, HTPs are used by 33.2% of the students aged 14–17 years.

The present study describes a nationwide case series of accidental ingestion by children of the metal-blade-containing HTP sticks, as reported in the medical database of Niguarda Hospital in Milan, Italy.

## METHODS

The European project Joint Action on Tobacco Control (JATC) 2^11^ conducted a study on the reporting of adverse health incidents after the use of electronic cigarettes and novel tobacco products among EU Member States. The study was part of Work Package 7, ‘Electronic cigarettes and novel tobacco products evaluation’.

The findings and needs on this topic were discussed in a webinar held in October 2023 entitled ‘Reporting on the health incidence after use of novel tobacco or nicotine products in European countries: Towards a harmonized approach’. The webinar revealed that there is currently no harmonized approach for the registration of adverse health incidents following the use of novel tobacco products and electronic cigarettes in Europe, nor is there a centralized collection of this information. The Italian National Institute of Health (ISS) contributed to the survey by contacting the Milan Poison Control Centre (PCC) of Niguarda Hospital (Italy) for data collection. The findings from the survey were presented during the webinar^12^.

Subsequently, between July 2023 and February 2024, a case series study of accidental ingestions by children of the metal-blade-containing HTP sticks was conducted by the Milan PCC, with the objective of collaborating with the aforementioned task of Work Package 7.

The Milan PCC is a 24-hour emergency service that provides consultancy and offers specialist advice for cases of acute intoxication throughout the country. The PCC offers advice to the Emergency Department of the Niguarda Hospital and can be contacted by both private individuals and health service personnel in the event of a suspected poisoning incident. All requests handled by the toxicologist are recorded in a computer database, which can be extracted for epidemiological purposes.

The data from the cases of accidental ingestion were subjected to analysis in order to identify the age distribution, clinical presentation and symptoms, diagnostic procedures performed, and medical management.

It is crucial to highlight that the sticks were only identifiable as blade-containing HTPs from July 2023 onwards. It is therefore not possible to rule out the possibility that previous cases recorded in the medical database as ‘electronic cigarettes’ or non-blade-containing HTPs may, in fact, include these specific new products, given that the product’s introduction to the Italian market occurred as early as December 2022.

In response to the clinical issue raised by the Milan PCC, the Italian Ministry of Health requested that the Italian National Institute of Health (ISS) verify the safety of the product and the actual hazard posed by the sharp metal parts in the sticks. Furthermore, ISS was asked to assess the clarity, visibility and size of the warnings on the packaging containing the sticks.

## RESULTS

A total of 40 affected patients were identified from 1 July 2023 to 29 February 2024. The majority of these patients were infants (aged 2–12 months) and toddlers (aged 1–4 years). The gender distribution was 50% female and 50% male, with a mean age of 12.3 ± 3.3 months (Table 1). Of the 40 patients, 33 (82.5%) were hospitalized. Of these, 29 underwent abdominal X-rays, and 24 X-rays were positive for the presence of the metal blade. Sixteen patients (40%) exhibited symptoms, including fifteen with repeated vomiting episodes (ranging from three to six episodes) and one with tonsillar pillar and genian mucosa cut lesions. In four cases, the blade was expelled through vomiting.

**Table 1:** Case series study: description and characteristics of the patients (infants and toddlers, mean age 12.3 ± 3.3 months), as reported in the medical database of the Milan Poison Control Center, 2024 (N=40)

*Case*	*Age (months)*	*Gender*	*Symptoms*	*Management*	*Notes*
1	9	F	None	Hospitalization, X-ray	
2	14	F	Vomiting	Hospitalization, X-ray	
3	8	M	Vomiting	Hospitalization	No X-ray, blade expelled through vomiting
4	12	M	None	Hospitalization, X-ray	
5	14	F	None	Hospitalization, X-ray	
6	12	F	None	Hospitalization, X-ray	X-ray negative, no blade
7	9	F	None	Hospitalization, X-ray	X-ray negative, no blade
8	9	M	None	No hospitalization	Blade found among clothes
9	9	M	None	Hospitalization, X-ray	
10	8	M	None	Hospitalization, X-ray, EGDS indicated	EGDS not carried out/not performed
11	15	M	None	Hospitalization, X-ray, EGDS carried out	Blade removed by EGDS
12	15	M	Vomiting	Hospitalization, X-ray, EGDS indicated	EGDS not carried out
13	10	M	None	No hospitalization	Blade ingestion excluded
14	20	M	Vomiting	Hospitalization, No X-ray	Blade expelled by vomiting
15	12	M	Vomiting	Hospitalization, X-ray	
16	14	M	Vomiting	Hospitalization, X-ray	
17	16	M	Vomiting	Hospitalization, X-ray, EGDS indicated	EGDS not removed blade because pylorus passed
18	13	M	Vomiting	Hospitalization, X-ray, Patient transferred to other hospital	No EGDS
19	11	F	Tonsillar pillar and genian mucosa cut lesions	Hospitalization, X-ray, Fibrolaryngoscopy	
20	10	M	None	No hospitalization	Blade found among clothes
21	9	F	Vomiting	Hospitalization, X-ray	
22	21	M	None	No hospitalization	Blade found among clothes
23	12	F	None	Hospitalization, X-ray	
24	10	M	None	Hospitalization, X-ray	
25	11	F	Vomiting	Hospitalization	No X-ray, blade found in the baby diaper
26	14	F	None	Hospitalization, X-ray	X-ray negative, no blade
27	12	F	None	No hospitalization	Blade found on the floor at home
28	11	F	None	Hospitalization, X-ray	
29	13	M	Vomiting	Hospitalization, X-ray	Blade expelled by vomiting
30	11	M	Vomiting	Hospitalization, X-ray	
31	8	F	None	No hospitalization	Ingestion excluded
32	8	M	None	Hospitalization, X-ray	EGDS considered but not performed
33	10	F	None	Hospitalization, X-ray	
34	13	F	Vomiting	Hospitalization, X-ray	
35	18	F	None	Hospitalization, X-ray	
36	16	F	Vomiting	Hospitalization, No X-ray	Blade expelled by vomiting
37	16	F	Vomiting	Hospitalization, X-ray	
38	13	M	None	Hospitalization, X-ray	EGDS considered but not performed
39	9	F	None	Hospitalization, X-ray	Suspected ingestion, X-ray negative, no blade
40	18	F	None	No hospitalization	Ingestion excluded

Two toddlers underwent an endoscopic procedure known as an esophagogastroduodenoscopy (EGDS). In certain instances, the surgeon attempted to remove the metal blade endoscopically, a procedure that required sedation and observation.

One infant sustained lacerations to the tonsillar pillar and genian mucosa, necessitating fibroscopy under anesthesia. In seven patients, the blade was discovered in the patient’s clothing, diaper, or on the floor of their residence, and hospitalization was not required, as ingestion was ruled out. Furthermore, the results of the ISS analysis on the product’s safety demonstrated the potential for accidental ingestion of the blade-containing stick, which was not adequately visible on the warning label on the packaging.

## DISCUSSION

Although the risks and toxicological management associated with exposure to tobacco and nicotine are well known, the cases reported in this study highlight an unexpected clinical issue related to the accidental ingestion of the sharp metal blade contained in HTP sticks.

The patients treated by the PCC were infants and toddlers in an age group in which they were unable to indicate whether they had ingested the tobacco sticks and whether they had symptoms that were not detectable on clinical examination. Consequently, regardless of the certainty of ingestion, the patients were required to be hospitalized in order to perform the necessary diagnostic procedures to determine the presence of the metal blade and its possible removal. It is widely acknowledged that X-ray exposure in pediatric patients should be undertaken with caution, given the potential risks associated with radiation^13^.

The decision to remove the blade endoscopically was dependent on a number of factors^14^. Primarily, it was crucial to ensure the correct localization of the blade within the esophagus or stomach. This required the availability of an endoscopist and anesthesiologist in the hospital with expertise in the emergency management of pediatric patients. Additionally, a careful assessment of the potential risks associated with sedation in each individual case was essential. The aforementioned risks may be influenced by a number of factors, including the presence of food in the stomach following a recent meal or the existence of chronic or acute diseases, as identified through a comprehensive clinical and anamnestic evaluation during the medical examination. Furthermore, the severity of the clinical manifestation upon admission to the emergency department must be carefully considered.

**Figure 1:**
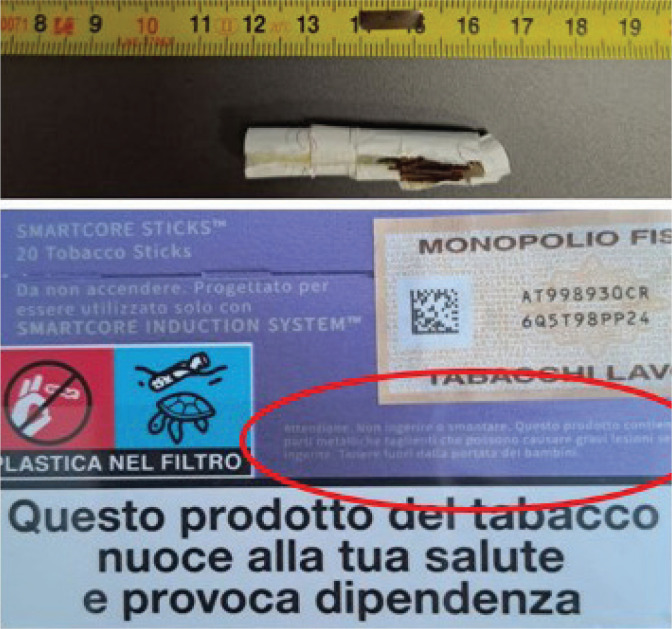
HTP sticks package almost illegible warning, at the time of the clinical alert raised by the Milan Poison Control Center (December 2023)

It is crucial to acknowledge that heated tobacco sticks are smaller in size than conventional cigarettes, which renders them more easily accessible and ingestible by infants and toddlers. The packaging of HTPs containing a metal blade indicates the presence of the blade in small, almost illegible letters, which may contribute to a lack of awareness of the risk. Each individual package has a 0.5 cm × 4 cm warning label, which reads: ‘Caution. Do not ingest or disassemble. This product contains sharp metal parts that can cause serious injury if ingested. Keep out of reach of children’ (Figure 1).

In the event of ingestion, even if there is only a suspicion that both tobacco and the metal blade have been swallowed, the child must be hospitalized and undergo appropriate tests to determine whether nicotine poisoning or cut injuries are present.

Notwithstanding the potential dangers of the blade, incidences of lacerations were documented in one case, involving a child who had vomited the blade. It is possible that in other cases the blade remained trapped in the casing of the heated tobacco stick or the gastric mucosa was protected by the presence of food.

The exact cause of the gastric symptoms remains unclear, though it is possible that they were caused by nicotine or gastric irritation from the blade. However, it is important to highlight that one of our patients had cut injuries to the oral cavity directly attributable to the sharp edges of the blade.

Two components of the HTP sticks were identified as potentially dangerous: the tobacco filler, and the metal blade. The tobacco filler contains nicotine, an alkaloid with a stimulating effect on the central nervous system and the cardiovascular system, and an irritant effect on the stomach and intestines^15^. The blade is flat, thin, flexible, one centimeter long, and has sharp edges. From a toxicological perspective, the metal is not absorbable if ingested acutely and thus poses no risk from the material of which it is composed. However, the composition of the blade is not fully known, and it is unclear whether the induction heating process has resulted in any modifications.

According to the experience of the medical staff of the PCC, most of the emergency care department physicians were unaware of the presence of the blade in HTP sticks. In fact, they contacted the PCC only for information about treating potential tobacco poisoning. This resulted in a delay in the proper treatment of the patient and risk of overlooking potential internal injuries caused by the blade.

### Response policy actions

In response to a clinical issue raised by the Milan Poison Control Center, the Italian Ministry of Health’s Prevention Department commissioned an analysis of blade containing HTP samples on 5 December 2023. The objective was to ascertain the actual hazard posed by the sharp metal parts present in the sticks and to evaluate the adequacy of the warnings in terms of clarity, visibility and size.

On 18 December 2023, the Ministry of Health, in response to the data presented in the PCC report, issued an alert to draw attention to the presence of such metal parts and the risks associated with ingestion by young children or individuals with cognitive disabilities. The alert was disseminated to regional health authorities, the Italian Society of Emergency Medicine, the Italian Society of Pediatrics, the Italian Society of General Medicine and Primary Care, the Italian Federation of General Practitioners, and to all Poison Control Centers^16^. The same alert was disseminated throughout the JATC 2 network to inform EU Member States about this health issue.

**Figure 2:**
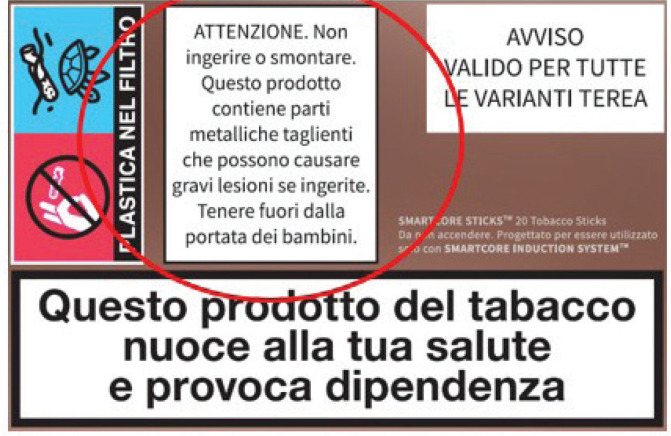
Modified more visible warning, after the corrective measure by the Italian Ministry of Health (Decree of March 2024)

Following the analyses conducted by ISS, on 15 March 2024, the Prevention Department of the Ministry of Health imposed new, larger, and more visible warnings on the package (Figure 2), which were to be implemented within two months. The new warning must cover approximately 19% of the reverse side of the package. In the interim, the manufacturer was obliged to disseminate the revised warning to retailers, who were required to affix it in front of the shelf with the blade-containing HTPs^17^. This was a provisional solution for the disposal of old packaging. As of June 2024, the new packages with larger warnings are available on the market.

It is recommended that regulators consider banning HTPs containing metal blades, or at least require warnings of larger sizes on the packages of these products, in order to protect the health of the pediatric population and other vulnerable groups.

### Strengths and limitations

It is important to note that it was challenging to identify all the cases related to blade-containing HTPs among those involving electronic cigarettes and tobacco products. Indeed, over the years, tobacco derivatives have been classified generically by category in the PCC database (tobacco, cigarettes, nicotine, electronic cigarettes, etc.). The coding of this specific product was only recently introduced. Moreover, the choice of coding the product presents two critical issues: accurate identification by the individual requesting the consultation and adequate coding by the toxicologist carrying out the consultation. It is also important to consider that the emergency room physician, who is already experienced in the management of tobacco poisoning, may not contact a PCC if they are unaware of the presence of the blade.

In light of these considerations, we believe that the number of the reported cases in this study is underestimated. However, it is sufficient to highlight an emerging clinical issue and take corrective actions to limit health risks for consumers and little children.

## CONCLUSIONS

The Ministry of Health disseminated an alert, about the potential dangers of metal-blade-containing HTP stick ingestion, to regional health authorities, the Italian Society of Emergency Medicine, the Italian Society of Pediatrics, the Italian Society of General Medicine and Primary Care, the Italian Federation of General Practitioners, and all Poison Control Centers. The observed clinical cases required immediate action to mitigate health risks, particularly given that exposure primarily affected the pediatric population. Modifying the packaging with more visible warnings and raising awareness among both the public and medical practitioners, as the Ministry of Health did, is crucial to prevent future cases and ensure a prompt and informed response in emergency situations.

It is imperative that public health institutions implement information campaigns and corrective actions to reduce the risk associated with the ingestion of these products. It is of the utmost importance to raise awareness among the general public and medical professionals, particularly pediatricians and emergency department physicians, about the potential dangers of metal-blade-containing HTP stick ingestion by the pediatric population and individuals with cognitive impairment.

## Data Availability

The datasets used and analyzed during the current study are available from the corresponding author upon reasonable request.
